# Study on Mechanical and Acoustic Emission Characteristics of Backfill–Rock Instability under Different Stress Conditions

**DOI:** 10.3390/s24134112

**Published:** 2024-06-25

**Authors:** Longjun Dong, Mingchun Yan, Yongchao Chen, Longbin Yang, Daoyuan Sun

**Affiliations:** School of Resources and Safety Engineering, Central South University, Changsha 410083, China; lj.dong@csu.edu.cn (L.D.); yanmch@csu.edu.cn (M.Y.); y.c.chen@csu.edu.cn (Y.C.); lbyang@csu.edu.cn (L.Y.)

**Keywords:** acoustic emission, sensor network, mechanical property, damage evolution

## Abstract

Unveiling the mechanical properties and damage mechanism of the complex composite structure, comprising backfill and surrounding rock, is crucial for ensuring the safe development of the downward-approach backfill mining method. This work conducts biaxial compression tests on backfill–rock under various loading conditions. The damage process is analyzed using DIC and acoustic emission (AE) techniques, while the distribution of AE events at different loading stages is explored. Additionally, the dominant failure forms of specimens are studied through multifractal analysis. The damage evolution law of backfill–rock combinations is elucidated. The results indicate that DIC and AE provide consistent descriptions of specimen damage, and the damage evolution of backfill–rock composite specimens varies notably under different loading conditions, offering valuable insights for engineering site safety protection.

## 1. Introduction

With the gradual increase in mining and consumption speed of mineral resources, surface and shallow ore bodies are being depleted due to long-term mining activities. Consequently, underground mining of mineral resources has become mainstream, progressively moving to greater depths [1,2]. As mining depth increases, ground stress rises, leading to increasingly complex mining conditions. Frequent mining activities result in extensive goaf formation, altering the rock mass from its initial three-way load equilibrium state to bidirectional or unidirectional loading, thereby readjusting and redistributing energy storage within the rock mass [3,4,5,6]. This emphasizes the increased necessity for ground pressure management in deep mining. Filling mining technology has become the primary choice for most mines to mitigate various disasters and environmental pollution issues associated with mining activities. Filling mining technology offers clear advantages in fully recovering mineral resources, controlling ground pressure in deep mining, and enhancing environmental protection, yielding remarkable results [7,8]. However, in practical production activities, deformation of the backfill body [9] due to high stress, loss of false top backfill mortar, and frequent top sheet collapse significantly impact the safe production of deep mining. Additionally, the backfill body is subjected to a complex production environment within the stope and is influenced by blasting disturbance. During typical mining backfill processes, the backfill body gradually forms a relatively stable rock mass structure with the surrounding rock after a maintenance period following its placement in the stope. The effect of frequent mining operations on the combined structure of the backfill body and surrounding rock of the upper and lower walls presents a significant challenge to the safety measures in the stope. Hence, it is crucial to investigate the mechanical properties of the backfill–rock combination, identify the damage evolution law of the backfill–rock combination, and provide appropriate guidance for stope safety protection measures based on the findings. This is essential for enhancing the level of mine disaster prevention and control and mitigating stope production risks.

Currently, research on backfill–rock combinations primarily focuses on the mechanical properties of simple structural assemblies under uniaxial compression [10,11], interface damage behavior [12,13,14,15], and the impact of backfill characteristics on the overall structure [16]. Xin et al. [17] established a dual-structure shear constitutive model to reveal the full shearing deformation evolution features of geological material. Xu et al. [18] revealed the strength growth law of the FCF and its crack expansion law to further study the mechanical properties and failure characteristics of the composite load-bearing structure composed of the filling body—coal pillar—filling body (FCF) in the co-bearing area. These studies provide some ideas for studying mechanical properties and damage mechanism combinations.

DIC is a technique for analyzing the surface displacement and strain of specimens based on photographs taken during testing. DIC has been widely used in rock testing. Yang et al. [19] studied the deformation behavior, fracture development, and failure pattern of CPB using a digital image correlation (DIC) technique. Zhao et al. studied the damage distribution characteristics and strain field evolution law of the filling body under different blast loading strengths using the DIC method and fractal theory. Nevertheless, DIC’s monitoring area in complex test environments like biaxial or triaxial is restricted, leading to insufficient analysis of the specimen’s overall damage process.

Acoustic emission (AE) represents the elastic waves excited by sudden changes in the composition, such as cracking. Analyzing the AE signal parameters and pinpointing the AE source can enhance the description of the specimen’s internal damage process [20,21,22]. Dong et al. used the RA-AF to understand the comparative relation of different micro-fracture types [23] and proposed a velocity-free MS/AE source location method to meet the high-accuracy locating requirements in complex two-dimensional and three-dimensional hole-containing structures [24]. Liu et al. [25] investigated the rock samples using triaxial cyclic loading-unloading tests and acoustic emission monitoring. Meanwhile, previous studies have shown that the arrangement of sensors has a significant impact on positioning accuracy [26]. Li et al. [27] concluded that the sensor network does not introduce location errors but amplifies existing errors in input data during the source location process. They found that location accuracy decreases dramatically when the AE source is at the edge or outside the sensor arrangement. Dong et al. [28] further researched error distribution within the sensor network, discovering that the center of a rectangular network has a high-precision positioning area, while significant errors occur along the symmetry axis.

Currently, an increasing number of scholars are integrating AE and DIC techniques to provide a clearer description of the specimen’s damage process from various perspectives [29,30,31]. Zhao et al. [32] used the digital image correlation (DIC) and acoustic emission (AE) techniques to study the failure mechanisms of cement–fiber–tailings matrix composites (CFTMCs) in unconfined compression strength tests. Zhou et al. [33] analyzed the effects of static rates on the CPB compressive strength and failure characteristics of CPB with different static rates in real-time using acoustic emission (AE) and digital image correlation (DIC) techniques. Zhang et al. [34] used AE and DIC techniques to obtain a more accurate cracking load, the contribution of aggregate interlock, and the angle of the compression field. The result shows that the combination of DIC and AE can describe the damage process in more detail. Ashraf and Rucka [35] investigated the fracture evolution of different concrete specimens through the integrated application of two diagnostic techniques, AE and DIC, under three-point bending tests. The results indicate that the combined AE and DIC techniques are highly effective for the detection of damage and ductility performance in fiber-reinforced concrete structures. Zhang et al. [36] investigated the exterior and interior coalescence process of fawned rocks with AE and DIC techniques. The results shows that the exterior and interior fractures in microscopic and macroscopic scales can be identified from the combination of AE parameters and DIC results.

Compared to single fractal methods, multifractal methods use spectral functions to describe the fractal structure more precisely, capturing the volatility of the fractal object at different levels. Currently, many scholars have conducted research in this area. Kong et al. [37] elaborated on the time-varying multifractal laws of AE counts about coal loading. Zhang et al. [38] used multifractal to study the waveform characteristics of red sandstone under uniaxial compression and found that its combination with frequency analysis can more accurately identify rock failure in engineering practice.

This work focused on backfill–rock combinations as the subject of investigation. We analyzed the surface displacement and strain evolution process under various loading failure paths, as well as the variation rules of acoustic emission characteristic parameters and multifractal parameters. Additionally, we explored the mechanical properties and damage mechanisms of backfill–rock combinations from both macroscopic and microscopic perspectives. On the macroscopic level, we directly observed the damage evolution process of the specimens at different loading stages using DIC technology, while on the microscopic level, we studied the internal rupture source information through AE technology, integrating the location results of AE events.

## 2. Backfill–Rock Specimens and Testing System

**Figure 1:**
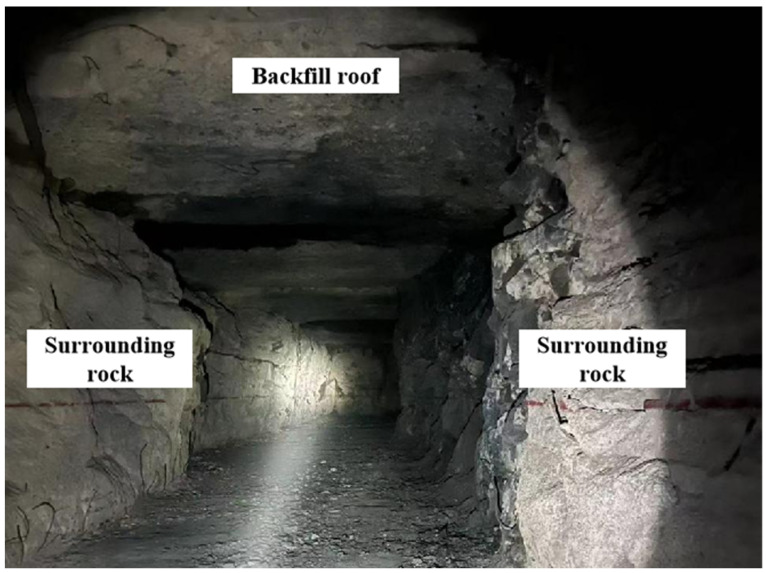
Field engineering structure about backfill–rock.

To investigate the instability mechanism of the backfill–rock approach structure, composite specimens with corresponding proportions of backfill and rock were prepared. Biaxial compression tests were conducted under various loading conditions to simulate the damage evolution of the stope approach structure under abnormal stresses from the roof or two sides in actual engineering conditions. The granite used in the composite for the biaxial compression test in this study was extracted from the surrounding rock of the 1018 middle section of a metal mine in Jinchang City, Gansu Province. The overall structure of the specimens is consistent with the field condition (Figure 1). The surface of the utilized granite is smooth and flat, devoid of macroscopic joint cracks and weak structural planes. Additionally, to ensure comparability of the test results and minimize the influence of accidental variations, granite specimens were extracted from the same direction of the same rock block. The backfill in the composite specimens is proportioned identically to the backfill used in the artificial filling process of the second mining area of Jinchuan Company in Jinchang City, Gansu Province. It consists of cement, aggregate, and water mixed in specific proportions with the cement provided by the company.

The filling aggregate serves as the primary material in the backfill, akin to the sand and stone in concrete, forming the backbone of the backfill structure. The downward-approach filling mining method necessitates the uniaxial compressive strength of the backfill to attain 1.5 MPa, 2.5 MPa, and 5 MPa at 3 days, 7 days, and 28 days, respectively. Based on mine experimentation and field application, the backfill aggregate is determined to comprise −3 mm rod scrub sand and Gobi sand. The aggregate configuration of the backfill in this study aligns with this determination. To maintain test material consistency, the backfill of the composite specimens adheres to the ratio utilized in the actual mining backfill process. Table 1 presents the specific composition of the backfill part in the composite specimen of this study.

The specimen size is 150 mm × 150 mm × 150 mm (length × width × height), and the reserved hole position is in the middle of the whole specimen. The size is 50 mm × 150 mm × 50 mm (length × width × height). Prior to specimen preparation, a custom mold was fabricated based on the specimen dimensions, with a foam block used to create an empty space at the center of the composite specimen.

**Figure 2:**
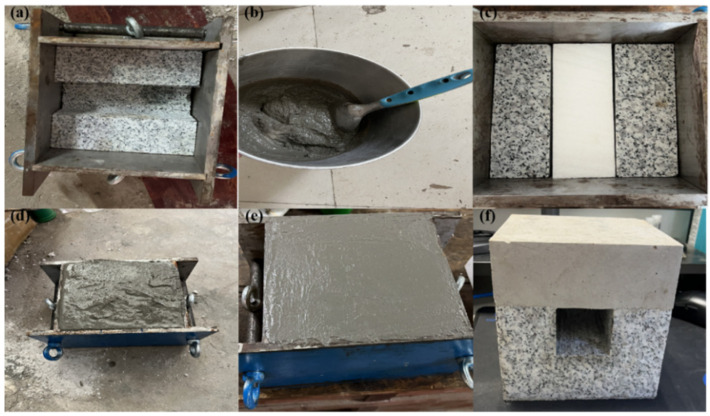
Preparation steps for the composite specimens: (**a**) Mold preparation, (**b**) Preparation of backfill materials, (**c**) Place a foam block to reserve the hole, (**d**) Pour the backfill materials into the mold, (**e**) Smooth the surface, (**f**) Complete demolding.

Before pouring the composite specimens, lubricating oil was applied to the inner mold wall and foam block surface to ease demolding. During pouring, the foam block is initially positioned in the pre-made stope approach location, followed by the mixing and uniform stirring of cement mortar according to the specified ratio. The mixture is then poured into the mold, with repeated vibration to remove air bubbles and smooth the surface in a timely manner, ensuring a relatively smooth and flat surface. After 24 h of pouring, the specimens were de-molded and transferred to a standard curing room maintained at 20 ± 2 °C and 90% humidity for 28 days. Prior to experimentation, the surface of the backfill component was smoothed further using sandpaper. The primary preparation steps for the composite specimens are illustrated in Figure 2.

This study employs the YJW-20000S micro-computer-controlled electro-hydraulic servo large-scale rock three-way five-sided loading test system (Jointly developed by Central South University and Shanghai Hualong Testing Instrument Co., Ltd., Shanghai, China). The test system offers three-way loading modes categorized into load control and displacement control. The load-indicating value error is within ±1%, and displacement measurement accuracy exceeds ± 1%, with a resolution of 0.001 mm.

**Figure 3:**
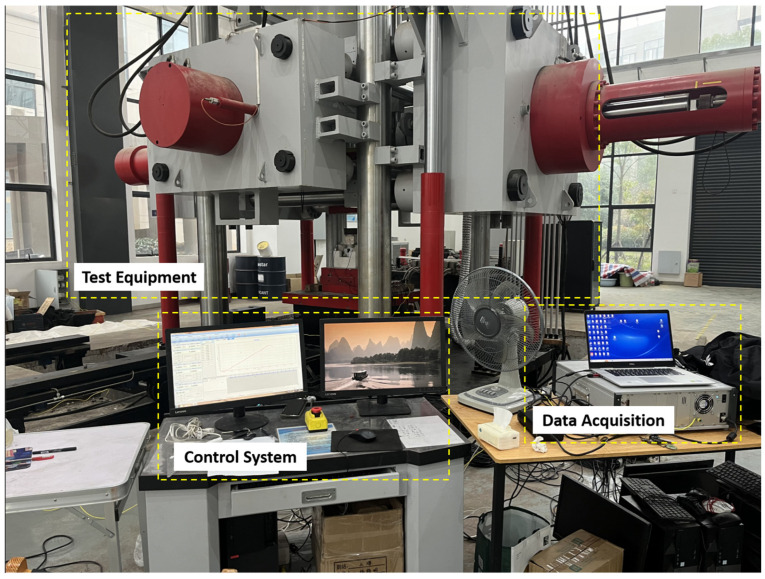
Different test systems.

The AE monitoring equipment utilizes the ASMY-6 acoustic emission system with VS45-H piezoelectric sensors (From Vallen, Wolfratshausen, Germany), which are single-component sensors and the frequency range of AE signal recording is 25–450 kHz. The AE system is configured with a sampling frequency of 10 MHz, considering environmental noise, with a test threshold set at 52 dB. The loading system and AE system are shown in Figure 3.

**Figure 4:**
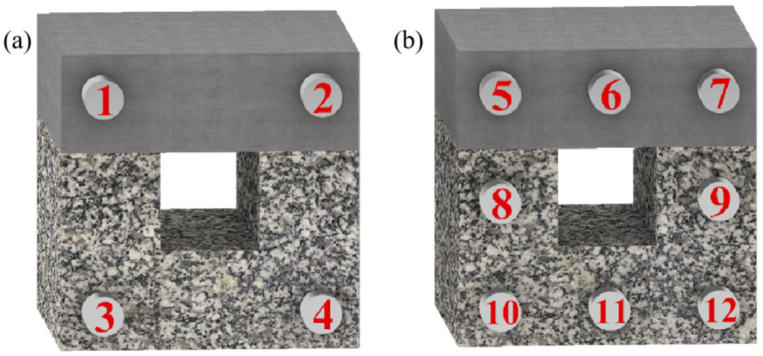
AE Sensors layout: (**a**) front; (**b**) back.

Twelve AE sensors are employed. The sensor grid is arranged in a rectangular pattern on the typical surface position of the combination, forming a sensor monitoring network. Due to the need for DIC monitoring on the front, four sensors are placed on the front, while eight sensors are positioned on the back, as illustrated in Figure 4.

**Figure 5:**
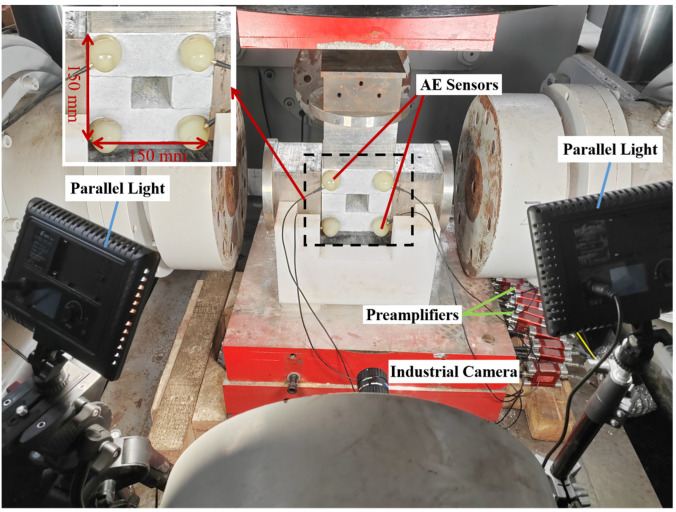
AE system, DIC system and the size of the specimen.

The test employs a ME2P-1230-23U3M industrial camera (Daheng Imaging Technology Co., Ltd., Beijing, China) for speckle imaging, with an image size of 4096 × 3000 pixels and a frame rate of 5 frames/s. Throughout the test, the specimen surface’s monitoring area is illuminated to guarantee clear speckle photo capture by the camera. The AE system and DIC system are shown in Figure 5. Before the experiment began, measures were taken to adjust the position and angle of the fill light, as well as the focus and position of the camera, to ensure that the photos taken in the software were of high quality. In the subsequent processing of speckle photos, high-quality speckle images were also selected for analysis.

Prior to the testing initiation, a layer of white matte paint was evenly sprayed onto the front surface of the composite specimens to conceal particles and structural planes, mitigating their influence on subsequent speckle analysis. Subsequent to allowing the specimens to stand for a period, the dried white matte paint on its surface, a black matte paint is sprayed diagonally upward at a distance, evenly sprinkling it onto the white matte surface to create speckles. Following natural drying, the subsequent test is conducted. Prior to commencing the test, a prestress of 2 kN was applied to the composite specimens to achieve complete contact with the loading block. The instantaneous stress acting on the specimen when the loading block contacts it generates AE signals considered as noise. Therefore, we applied prestress to the specimen before recording AE data to ensure full contact and eliminate this interference. To expose a complete surface for DIC monitoring, we must use either biaxial or uniaxial testing. Given the on-site conditions, the surrounding rock is in a typical two-dimensional stress state when a roadway or mining area is excavated. And researches have shown that the difference of stress in different directions causes the anisotropy of rock fracture and thus leads to the obvious anisotropic characteristics of wave velocity variations [39,40]. Therefore, we chose biaxial testing. Two loading methods were employed in the test. The first method involved simultaneous loading of the *Y*-axis and *Z*-axis of the loading system at 500 N/s. Once the *Z*-axis stress reached 5 MPa, it stabilized, while the *Y*-axis continued increasing until real-time monitoring showed a drop to 80% of peak stress. At this point, the specimens halted automatically, thereafter becoming destabilized and destroyed via axial stress. The second loading method mirrors the first during the pre-loading stage. Upon reaching 5 MPa, the *Y*-axis stress stabilizes while the *Z*-axis continues loading at the initial rate, resulting in specimen destabilization and damage due to horizontal stress.

## 3. Results

### 3.1. DIC Measurements Results

**Figure 6:**
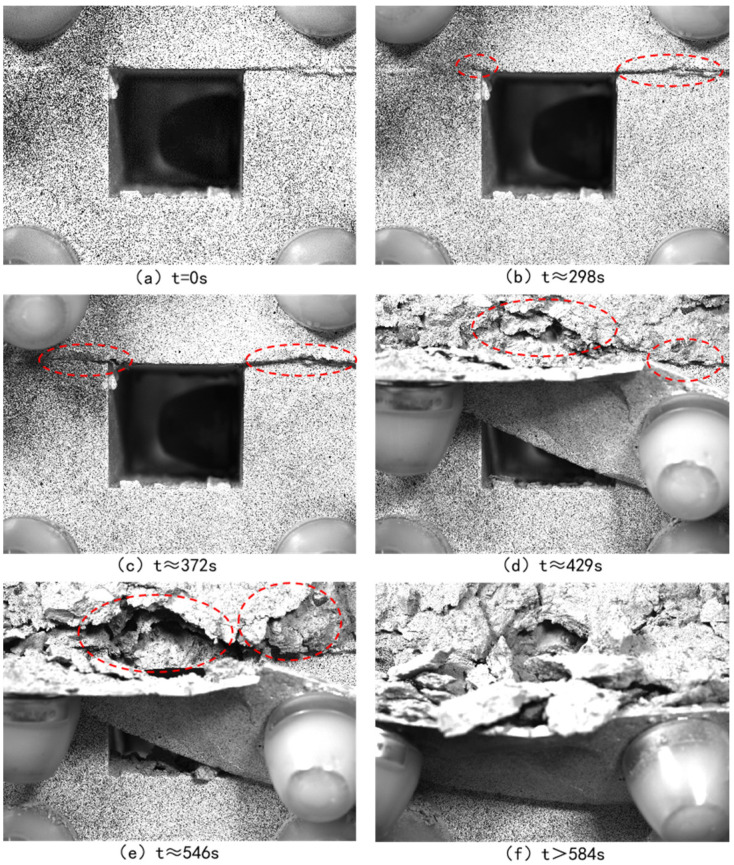
Speckle photos at different loading times during axial loading.

**Figure 7:**
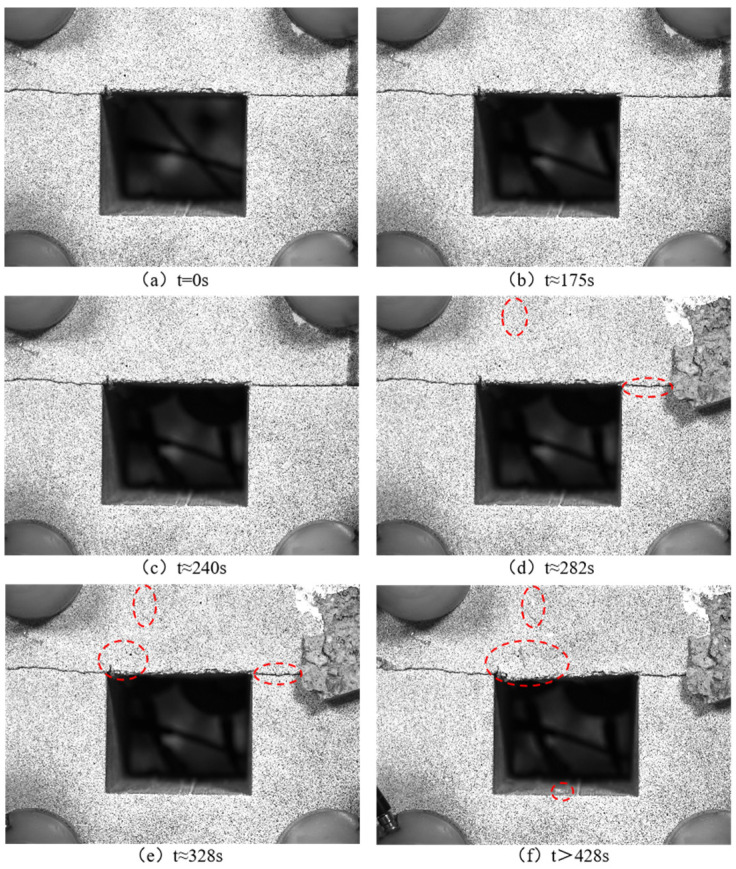
Speckle photos at different loading times during horizontal loading.

**Figure 8:**
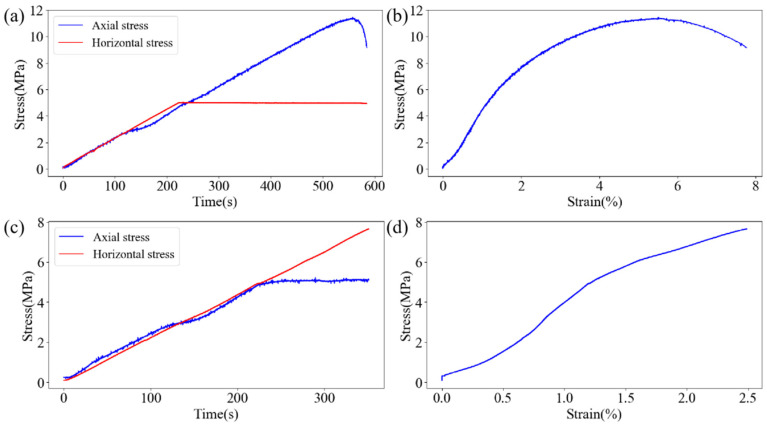
Stress–time and strain–stress curves under different loading conditions: axial loading: (**a**) stress–time, (**b**) stress–strain; horizontal loading: (**c**) stress–time, (**d**) stress–strain.

Figure 6 and Figure 7, respectively, depict the macroscopic damage process of specimens captured by DIC under various loading conditions. The corresponding stress–time and stress–strain curves are depicted in Figure 8.

The speckle photos clearly depict the damage evolution of the specimen at various loading times. Dislocation starts to manifest at the interface around 298 s of loading. At approximately 372 s of loading, noticeable deformation emerges at the combination interface. Concurrently, the filler material above the void area gradually stretches due to the applied force. Around 429 s of loading, numerous cracks become observable within the backfill material. With continued loading, the backfill progressively loses stability and eventually fails in a spalling manner above the void area, as illustrated in Figure 6.

Nonetheless, under horizontal loading conditions, the damage morphology of the specimens exhibited discernible variations in the speckle photos. At approximately 282 s of loading, deformation transpires at the interface, accompanied by minor cracks at the backfill’s apex. Upon reaching approximately 328 s of loading, the specimen continues expanding at the initial deformation site, initiating crack propagation tendencies along the left edge above the cavity. Continuous lateral loading and axial maintenance loading lead to the formation of a conspicuous shear failure crack along the upper cavity’s left edge.

#### 3.1.1. Strain and Displacement Fields under Axial Loading Condition

**Figure 9:**
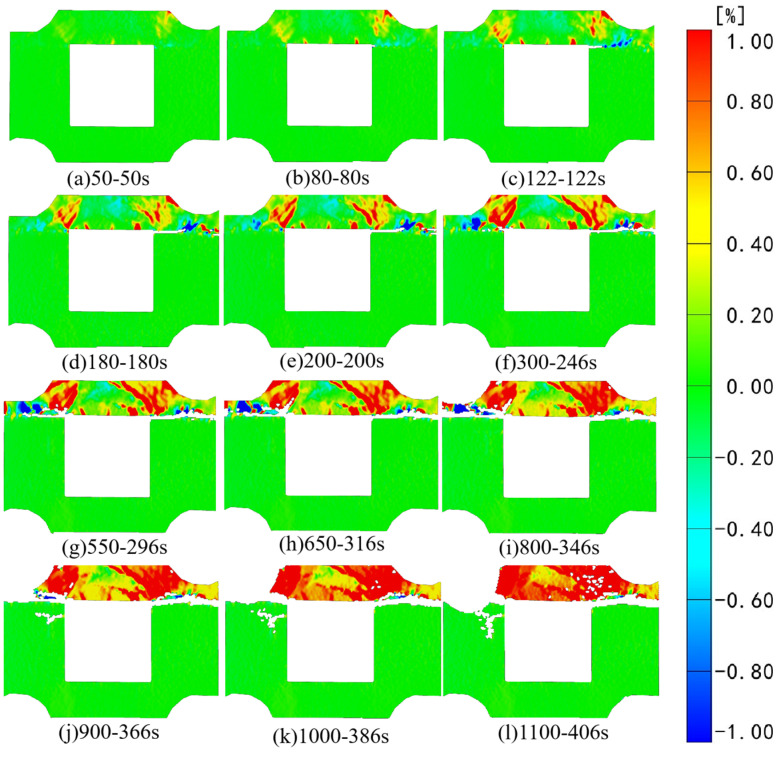
Strain field changes in the X direction under axial loading.

The strain and displacement fields in various directions are analyzed based on the initial speckle photos. To enhance digital speckle image processing efficiency, the images are screened while retaining essential information regarding specimen instability fractures. Figure 9 illustrates the results of strain field analysis in the X direction (aligned with the specimen coordinate system) during the loading process. Photo naming conventions in this study are based on the selected photo’s location and corresponding loading time. The figure reveals significant strain field changes in the X direction within the backfill before reaching a horizontal stress of 5 MPa. At 80 s of loading, tensile strain concentration occurs along the left and right endpoints above the goaf, while compressive strain concentration manifests at the right interface. At 122 s of loading, notable pressure and strain concentrations occur at the right interface, along with partial dislocation observed between the backfill and granite. At 296 s of loading, the initial tension strain concentration area in part of the backfill continues evolving. The dislocation area expand horizontally at the right interface, while partial pressure strain concentration occurs at the left interface. Figure 6b clearly depicts crack growth at the interface. This occurs because horizontal loading has ceased, with the composite specimen primarily subjected to axial stress. After 366 s of testing, the tension strain concentration area above the void continues expanding, with noticeable dislocation occurring at the combination interface, consistent with the findings in Figure 6c. Throughout the test, partial tensile strain in the composite backfill continues to evolve. It is noteworthy that throughout the loading process, the granite portion of the DIC monitoring surface remains relatively stable, with uniform strain distribution in each region.

**Figure 10:**
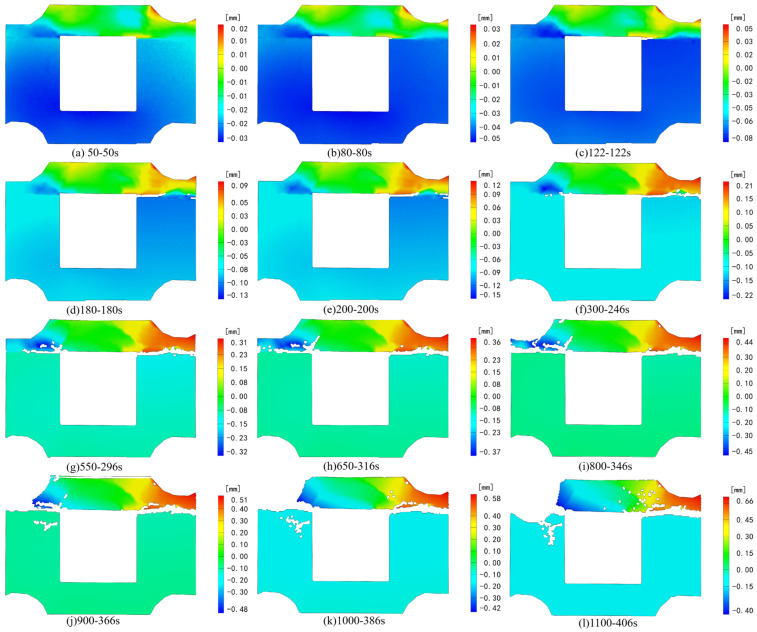
Deformation field change in the X direction under axial loading.

Following the time node of strain field change in the X direction, we chose the corresponding time node of deformation field changes (Figure 10) for analysis. At 50 s of loading, deformation emerges at the right interface of the combination near the cavity’s end, aligning with the results of strain field analysis. After 180 s of testing, noticeable deformation areas appear in opposite directions at the left and right ends of the backfill body. The discrepancy in deformation under the combination’s right-side interface indicates an interface dislocation phenomenon. At 296 s into the test, horizontal loading ceased, and the composite specimen primarily endured axial stress, yet prominent deformation remained concentrated at the composite backfill’s left and right ends. Throughout the subsequent loading process, the deformation zone progressively extended from both ends of the combination backfill towards the center, signifying the emergence of a tensile stress area above the void region, consistent with the strain field findings along the X direction.

**Figure 11:**
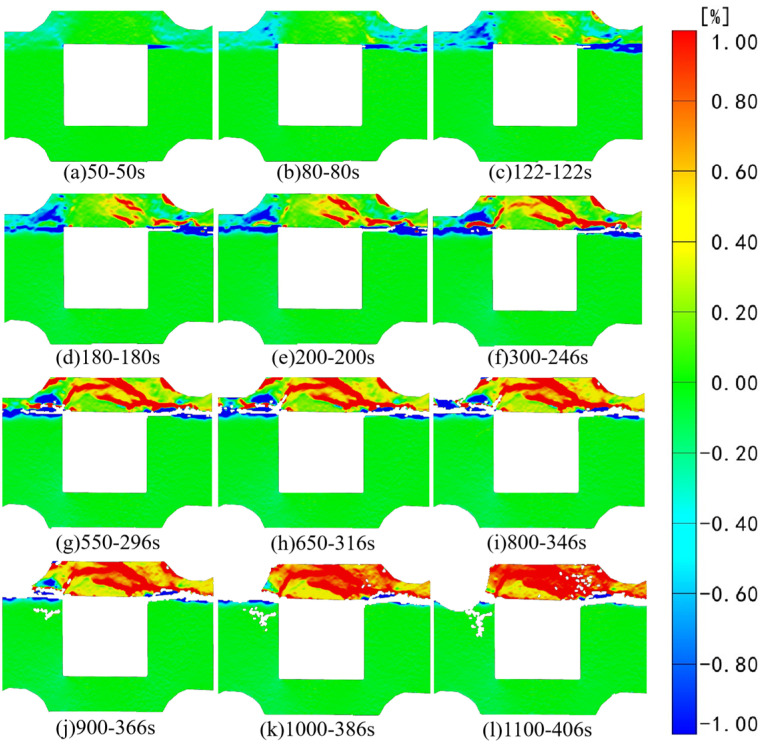
Strain field changes in the Z direction under axial loading.

Figure 11 depicts the strain field analysis in the Z direction, aligning with the Z direction of the specimen coordinate system, throughout the test loading process.

Initially, axial and horizontal loads increase simultaneously, leading to a compressive strain concentration area at the combination’s interface and the left and right endpoints above the cavity. By 80 s of gradual loading, tensile strain gradually develops in the backfill above the void area. Subsequently, around 122 s into the loading process, a blank area emerges at the combination’s right-side interface, signifying speckle tearing and further evolution of the tensile strain area in the backfill above the void area. By approximately 246 s into the loading process, the speckle at the combination’s right-side interface is fully torn, signifying significant deformation at this interface. In contrast, part of the tensile strain in the initial pressure strain concentration area at the combination’s left-side interface gradually evolves. Simultaneously, there is a notable concentration of tensile strain in the backfill above the void. Following significant deformation at the right interface, its support capacity diminishes, subsequently leading to the emergence and rapid evolution of a tensile strain concentration area at the left interface. At 296 s into the test, significant deformation also occurred at the left interface, resulting in the tearing of the surrounding speckle. Subsequently, in the strain field variation diagram, it becomes evident that the tensile strain concentration area of the backfill body above the cavity gradually extends to the entire area. Notably, in biaxial testing scenarios, the binding force along the backfill body above the goaf towards its left and right edges gradually strengthens, while the presence of the goaf grants the backfill body significant axial downward freedom. The alteration of binding force at both ends and the presence of the void area precisely result in the initial compressive strain in the Z direction of the backfill portion evolving into an arc-shaped tensile strain during loading, culminating in the instability and failure of the backfill portion.

**Figure 12:**
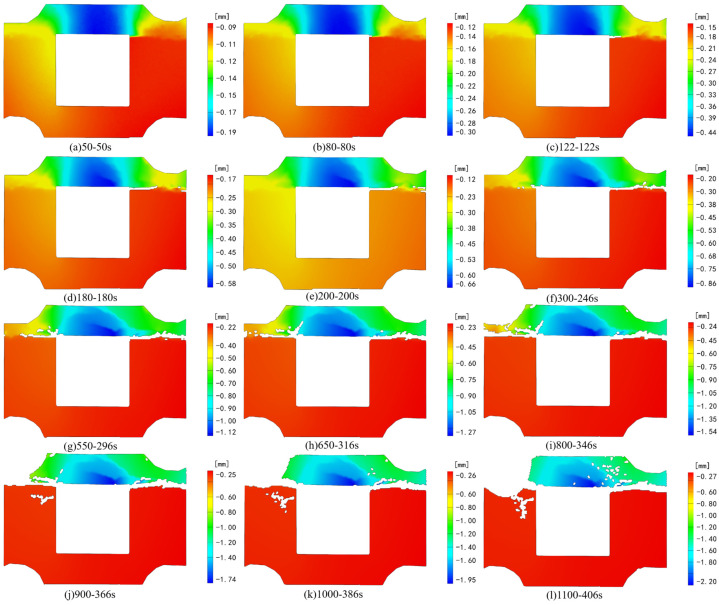
Deformation field change in the Z direction under axial loading.

Deformation in the Z direction of the specimen primarily occurs in the backfill above the cavity, with no noticeable displacement in the granite section (Figure 12). During test loading, deformation in the specimen under axial and horizontal dynamic loads primarily focuses on the backfill above the cavity. By 122 s into the loading process, significant deformation along the Z negative direction becomes evident in the backfill above the void under the dynamic load. With the binding force on the specimen’s horizontal direction gradually strengthening from the middle to the edge, deformation values decrease from the middle towards both ends. Upon reaching 200 s of continuous loading, significant deformation occurs at the combination’s right-side interface, accompanied by a weakening of the granite’s supporting effect on the right side. This leads to the deformation of the upper backfill in the negative Z direction, with the prominent deformation area above the initial backfill concentrating in the middle. At 246 s into the test, reaching the preset value, the horizontal load intensifies, further exacerbating deformation at the right interface. By 316 s of loading, due to the gradual intensification of deformation on the right side and the weakening support of the granite, deformation at the left interface accelerates, generating cracks along the edge of noticeable deformation disparity. With the continued increase in axial load, the entire composite backfill moves along the *Z*-axis in a negative direction, and the prominent deformation area of the specimen consistently concentrates on the backfill above the cavity.

#### 3.1.2. Strain and Displacement Fields under Horizontal Loading Condition

**Figure 13:**
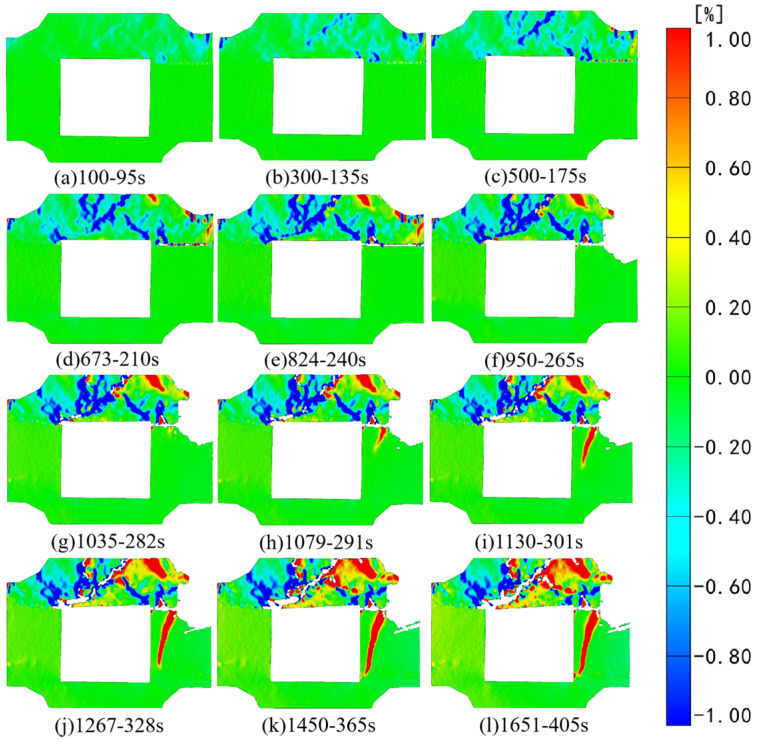
Strain field changes in the X direction under horizontal loading.

Figure 13 illustrates that during the initial loading stage, strain changes are primarily concentrated in the combination’s backfill. With the gradual shear failure of the backfill, a noticeable linear concentration area of tensile strain emerges downward from the interface in the right pillar of the bottom granite. At the same time, a minor evolution of tensile strain also occurs in the left pillar of the granite. At 95 s of loading, compressive strain concentration areas in the X direction start to manifest in the backfill, primarily at the upper right endpoint of the void area and the upper right sensor position. With ongoing loading, the compressive strain region undergoes further evolution, and both tensile and compressive strains simultaneously appear at the combination’s right-side interface, indicating the concurrent action of compressive and tensile stresses. The significant evolution of compressive strain from top to bottom is attributed to the constraints of axial load from above. This constraint gradually diminishes from top to bottom along the axial direction, causing the compressive strain evolution trend of the backfill above the goaf to transition from the top right to the bottom. In contrast, the compressive strain evolution at the left and right endpoints along the top of the goaf occurs simultaneously. At 240 s into the loading process, the speckle at the right interface completely tears, indicating deformation at this interface, and the supportive effect of the lower granite on the backfill weakened. During the attenuation of its supportive effect, the upper backfill experiences tensile strain generation alongside the continuous evolution of compressive strain. By 282 s of loading, the sensor on the top right of the specimen detaches, and cracks gradually emerge along the left endpoint on the upper side of the void, about 60 degrees to the right. Cross interactions between compressive and tensile strains could be observed around the cracks. As the test progresses, the backfill continues expanding along the initial crack, leading to regional strain concentration in the backfill beneath the crack. Notably, in the subsequent loading process, as the backfill part progressively undergoes shear failure along the crack direction, the granite on the right side transitions from the interface to a linear strain concentration area, while the granite on the left side exhibits a minor strain concentration.

**Figure 14:**
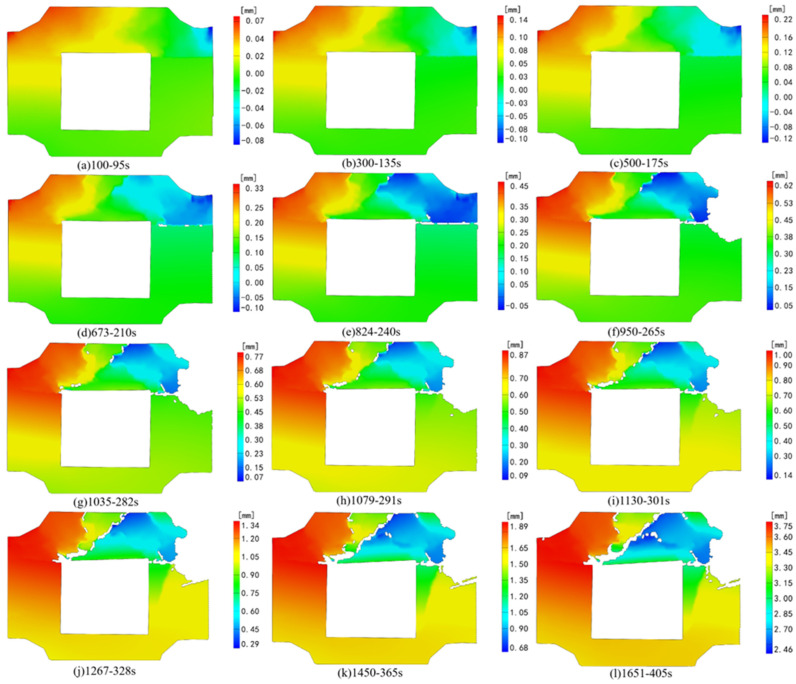
Deformation field change in the X direction under horizontal loading.

Figure 14 illustrates the analysis results of the deformation field in the X direction during the test loading. At 95 s of loading, the displacement of the filling body part of the combination specimen gradually decreases from left to right. In addition, due to the load on the right side, the upper right corner of the monitoring area shows negative displacement in the X direction. As the test progresses, a notable displacement difference emerges along the upper left end of the cavity. At 301 s into the test, obvious cracks develop in the original displacement difference area of the backfill body. As the top backfill body gradually destabilizes and fails, significant displacement differences appear on both sides of the linear direction in the right part of the granite. Throughout the loading process, continuous dynamic loading from left to right on the right side of the backfill body in the horizontal direction ensures that the displacement of the composite specimen in the X direction remains consistently positive once the axial load reaches the preset value, maintaining stability.

The deformation field in the X direction changes gently compared with the strain field in the X direction, and the regional gradient process occurs with the change of cracks in different stages of the composite specimen. The displacement of the backfill is relatively large compared with that of granite.

**Figure 15:**
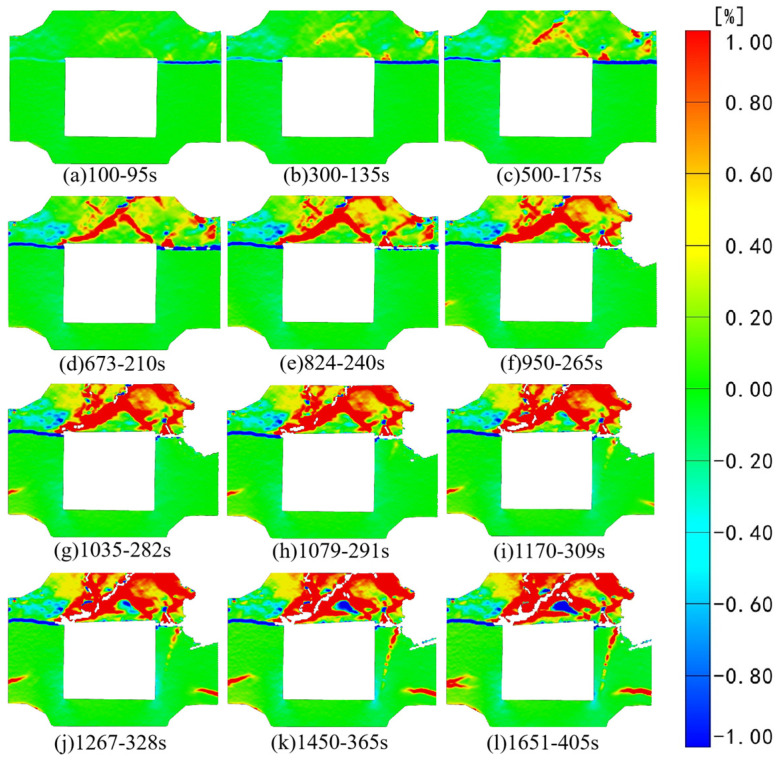
Strain field changes in the Z direction under horizontal loading.

Figure 15 presents the results of strain field analysis in the Z direction. Initially, at 95 s of loading, a significant Z-direction compressive strain concentration is evident at the right interface of the composite specimen, surpassing that at the left interface. Concurrently, the tensile strain region of the backfill body exhibits initial evolution. By 135 s of loading, the original strain area of the backfill body continued evolving, and a compressive strain concentration area gradually emerged in the left part of the backfill body. At 175 s of loading, the original strain region intensified and evolved, displaying a linear evolution trend from the left and right endpoints above the goaf to the middle part of the backfill body. This evolution path roughly formed a triangular shape with the upper edge of the goaf. As loading progressed, the tensile strain area of the backfill part rapidly intensified and evolved. Concurrently, cracks at the interface of the right side of the combination further developed, with the speckle essentially completely torn by 240 s of loading. At 265 s into the loading process, subtle cracks also develop in the original tension strain concentration area of the backfill body, leading to the speckle being torn and the sensor at the right upper end falling off. Notably, at this juncture, horizontal linear tensile strain is observed evolving at the left edge of the granite. This suggests that with the gradual instability of the backfill, the continuously increasing load increasingly impacts the granite part. Under this loading path, the left granite is affected before the right granite. With ongoing loading, some cracks in the backfill continued expanding, and the original tensile strain began exhibiting regional changes. At 309 s, it became apparent that the right backfill started exhibiting a negative linear tensile strain along the Z-axis from the interface, with a tensile strain region evolving from the right edge of the combination. As the experiment progresses, the strain area of the granite part undergoes rapid evolution, with Z-direction compressive strain concentration observable at the left and right ends below the void area.

**Figure 16:**
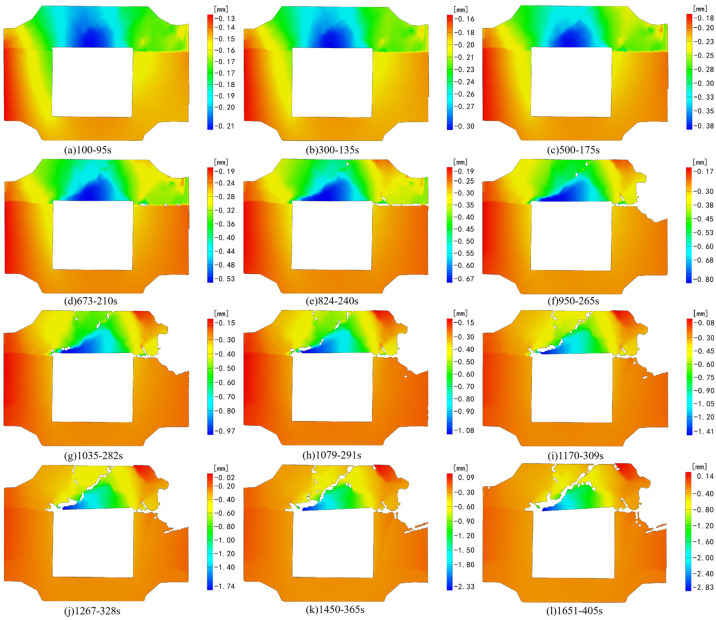
Deformation field change in the Z direction under horizontal loading.

In contrast to the Z-direction strain field, the deformation field of granite in the Z direction undergoes gentle changes. The deformation of the backfill part is noticeable throughout the entire process, while the deformation field of the granite part experiences some changes under initial stress, albeit minor overall. Figure 16 shows that the filling body above the void area exhibits the highest degree of freedom during the initial loading stage, displaying a relatively pronounced downward trend. By 210 s of loading, the negative displacement area of the backfill body above the void gradually diminishes downward, forming a triangular area as observed in the strain field. Concurrently, the displacement change of the granite part becomes gradually stable. With ongoing loading, cracks in part of the backfill body continue expanding, accompanied by evident deformation fields on both sides of the cracks. The degree of deformation gradually diminishes from the left endpoint above the void to the upper right along the trend of crack expansion, aligning with the changing trend post-failure of this part of the backfill body.

In the horizontal loading failure test, the instability failure process of the composite specimen under this condition is primarily divided into two stages. The first stage involves the instability failure of the backfill part. The second stage entails the stress deformation of the granite following the instability and failure of the backfill body. Upon the occurrence of instability and failure of the backfill part, the overall stress distribution of the composite specimen undergoes redistribution. During this period, the continuously increasing horizontal load notably affects the bottom granite, inducing a tendency of extrusion towards the empty area. At the left and right edges of the granite, a horizontal extension of the tensile strain linear concentration area occurs, while Z-direction compressive strain concentration can be observed simultaneously at the two ends below the void area. Simultaneously, due to the instability and rupture of the top backfill, the transfer path of the axial load alters. The combined effect of the right-side load forms a tensile strain concentration area from the interface to the right-side granite. Throughout the test process, the strain field in the monitoring area undergoes more pronounced changes than the deformation field, allowing for clearer observation of the dynamic changes in the granite part.

### 3.2. AE Measurements Results

#### 3.2.1. AE Source Location Method

The localization of AE sources can help us better analyze the distribution pattern of internal damage in the specimen. Therefore, we conducted localization calculations on AE events during the experimental process. In this process, we filter AE events using an arrival time difference threshold $∆t_{cr}$ and a triggering sensors threshold $N_{cr}$. For example, if the number of sensors triggered by a certain AE event is N, and N is greater than or equal to $N_{cr}$, and the arrival time difference between these N sensors satisfies $∆t_{cr}$, we consider it as a valid AE event. In this work, $N_{cr}$ is equal to 6. Accurate arrival time is crucial for the accuracy of AE source localization, and there are currently many arrival time picking methods available [41,42,43]. The pickup method we use is called the wavelet STA/LTA-AIC (WSA) picker. It combines the advantage of the wavelet transform technology, the ratio of short term average to long term average age (STA/LTA) method, and the Akaike information criterion (AIC). For all experiment results, the proposed WSA picker shows less location error compared with the AIC picker, regardless of geometry and source type [44].

The method we employed is an MS/AE source localization using an improved search algorithm in complex environments with GPU parallel computing (ISACE-GPU). Firstly, a specific grid model is established based on the structure of the positioning object. The smaller the unit length of the grid, the higher the positioning accuracy. Secondly, corresponding values are assigned to different grid points based on the wave velocity of different media. Once again, the theoretical arrival time from each sensor to each grid node is calculated, and one sensor as the reference sensor is selected to calculate the theoretical arrival time database. The observation data to the time difference database using the same method is calculated. The location in the grid node where the sum of squares of the time difference between each sensor and the source is minimized is considered as the localization result of the AE source.

#### 3.2.2. AE Source Location Results

**Figure 17:**
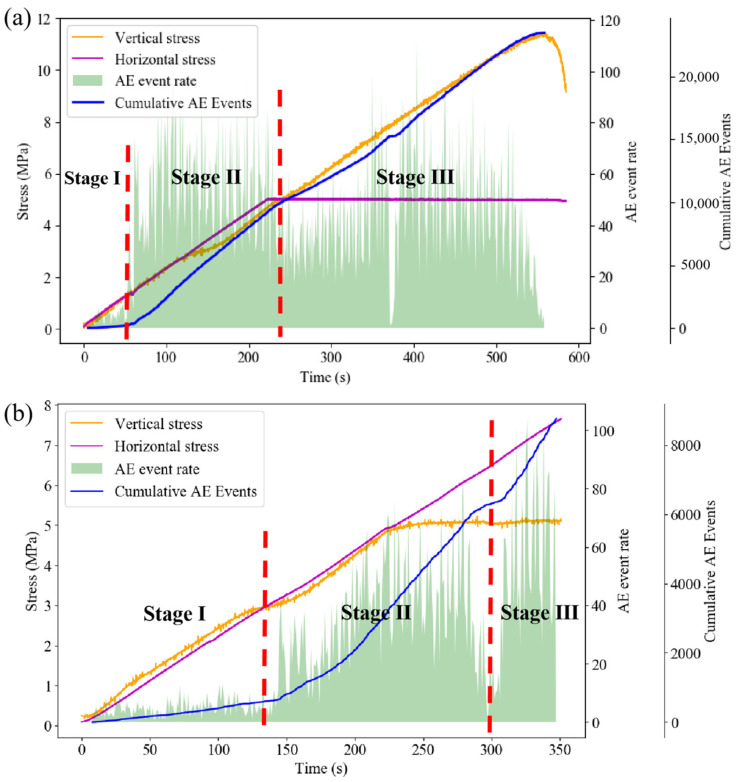
Time division based on AE event rate: (**a**) axial loading; (**b**) horizontal loading.

Indeed, DIC analysis exclusively reveals alterations on the monitored surface. To further explore the evolution process of internal specimen damage, AE events are monitored during the loading process. The loading process under different loading conditions is divided into three stages based on the variation trend of accumulated AE events (Figure 17).

**Figure 18:**
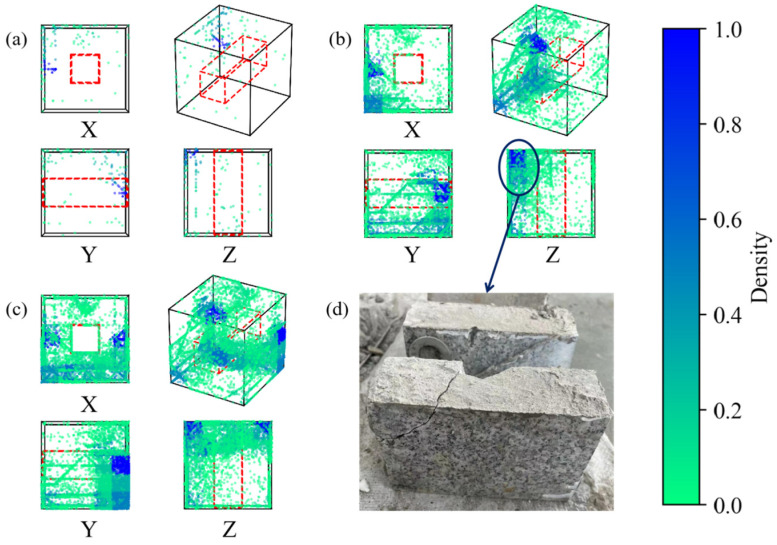
Distribution of AE events in different sections in different stages of axial loading: (**a**) Stage I; (**b**) Stage II; (**c**) Stage III, (**d**) macro failure.

The positioning results of AE events at different stages are depicted in Figure 18. During axial loading failure, AE activity in stage 1 remains minimal, primarily centered within the backfill and the left granite. During the second stage, as internal combination damage gradually evolves, acoustic emission events exhibit a pattern of rapid increase followed by a gradual decline. During this stage, noticeable abnormal strain concentration areas appear in the backfill within the strain fields of various directions within the DIC monitoring area. In the third stage, significant differences are observed in the distribution of AE events. AE events are concentrated above the void, with a high-density event concentration area observed in the granite part to the right of the void, and high-density events mainly occurring in the rear of the combination. After stopping the loading, an obvious large-scale rupture is observed in the granite part on the left rear side of the combination specimen, as depicted in Figure 18.

**Figure 19:**
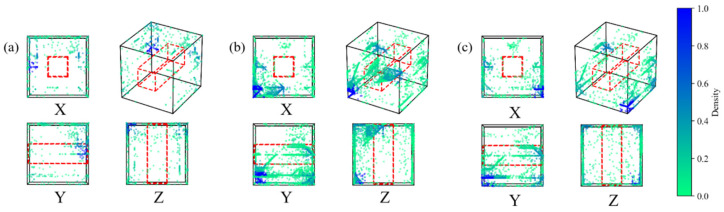
Distribution of AE events in different sections in different stages of horizontal loading: (**a**) Stage I; (**b**) Stage II; (**c**) Stage III.

During horizontal loading failure, acoustic emission events are initially concentrated on the top backfill and granite on both sides of the void during the initial compaction stage (Figure 19). Later, a few abnormal strain concentration areas appear in the top backfill. In the second stage, there is a rapid increase in cumulative AE events, mainly distributed in the left granite area. The AE events in the top pack transition from uniform distribution to being concentrated in the area above the void, and there is significant AE event accumulation on the rear side of the entire combination. Notably, significant abnormal strain concentration appears at the left granite edge in the late stage, which is consistent with the clustering area of acoustic emission events. In the third stage, AE events are more concentrated above the void in the X direction and evenly distributed in the Y direction. This indicates that the damage to the backfill body had evolved in most areas at this time. Moreover, the high-density AE event area in the granite part shifted from the left side to the right side, which is consistent with the abnormal strain concentration area presented by the granite on the right side.

#### 3.2.3. AE Characteristic Parameters Analysis

**Figure 20:**
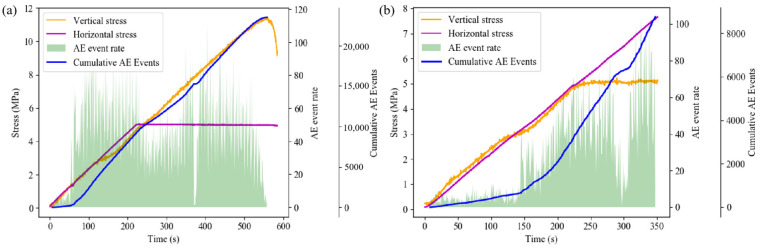
Variation of AE event rate and cumulative event number under different loading conditions: (**a**) axial loading; (**b**) horizontal loading.

Figure 20 illustrates the variation trends of the AE event rate and the cumulative AE event number of the composite specimen under different loading conditions over time.

Under axial loading failure conditions, the overall AE event rate exhibits a two-stage change trend of “increase—decrease—increase—decrease”. At the initial loading stage, internal micro-cracks and interfaces of each part of the composite specimen are compressed and sealed, resulting in a high-AE event rate and rapid increase in accumulated AE events. As the horizontal stress reaches the preset value, the granite part tends to stabilize, leading to a gradual decrease in the AE event rate. The composite specimen is approximately in the elastic deformation stage, resulting in a low-AE event rate. Subsequently, there is an obvious upward trend in the AE event rate, and the backfill part of the specimen rapidly transitions from the crack development stage to the stage of unstable crack expansion, leading to intensified internal damage and noticeable deformation. Simultaneously, a large-scale fracture occurs in the granite at this stage.

Under horizontal loading failure conditions, the AE event rate during the loading process exhibits a trend of “increase—decrease—increase”. Initially, the AE event rate is low, and the cumulative AE events increase gradually. Subsequently, there is a sudden increase in the AE event rate, mainly due to crack propagation inside the backfill, which manifests as rapid and concentrated strain evolution in the strain field. After the axial stress reaches the preset value, the AE event rate remains high for a period of time, gradually decreases, and then rapidly increases in the late loading stage. During this process, the damage to the backfill intensifies continuously, leading to the appearance of obvious cracks. As the backfill body part becomes unstable and fails, micro-cracks in the granite part begin to appear, resulting in strain concentration at the edge of the granite in the strain field. As large-scale cracks emerge in the backfill, the AE event rate gradually decreases. At this point, stress is concentrated in the granite part, leading to the rapid closure of micro-cracks in the granite and the gradual formation of internal damage, resulting in the rapid increase in the AE event rate in the final stage.

The energy variation during axial loading failure differs significantly from that during horizontal loading failure. Under axial loading failure conditions, the accumulated AE energy exhibits a step-like change. Initially, due to fewer AE events, the accumulated AE energy remains low, then increases rapidly with the increase in AE event rate. After the horizontal stress reaches the preset value, the rate of increase in AE cumulative energy decreases slightly, then increases with the increase in AE event rate and the occurrence of high-energy events until the axial stress reaches its maximum value. Notably, in this process, large energy events are mainly concentrated after the horizontal stress reaches the preset value.

**Figure 21:**
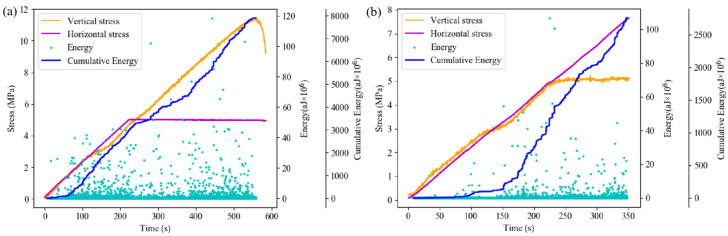
Variation of AE energy and cumulative energy under different loading conditions: (**a**) axial loading; (**b**) horizontal loading.

Under horizontal failure conditions, the accumulated acoustic emission energy initially shows a flattening trend before rising rapidly, with both accumulated energy and event energy at a low level during the initial loading stage (Figure 21). As the acoustic emission event rate gradually increases, events with larger energy begin to occur, leading to a rapid increase in accumulated acoustic emission energy. In the late loading stage, the AE event rate increases significantly, but the AE event energy remains at a medium level, with no occurrence of large energy events, corresponding to the compression stage of the granite at this time.

### 3.3. Multifractal Analysis

Due to the heterogeneity and anisotropy of rock materials, crack evolution under load is a nonlinear, unstable, and multi-scale process. Fractal theory, proposed by Mandelbrot [45], has been widely used in studying phenomena exhibiting self-similarity and nonlinearity. However, describing some complex research objects with a single fractal is overly simplistic, and it is more accurate to characterize them using multifractals. Acoustic emission signals generated by rock mass fracture are typical nonlinear signals that exhibit multifractal characteristics in both time and space scales [46]. Therefore, using the multifractal method to analyze the acoustic emission signal generated by rock mass failure and instability can provide a more accurate description of the fracture process. This work employs the multifractal detrended wave method (MF-DFA) to analyze certain acoustic emission characteristic parameters.

The AE signal energy and rise time of backfill–rock composite specimens under various stress conditions were analyzed using multifractal analysis, revealing distinct multifractal characteristics. The energy of AE signal corresponds to the intensity of rock fracture event. The greater the energy of AE signal, the higher the intensity of the corresponding rock fracture event, which is usually manifested as large-scale fracture. The AE signals produced by tension rupture have the characteristics of short rise time, and those produced by shear rupture have the characteristics of long rise time. The multifractal spectrum curve exhibits a single-peak bell shape, attributed to the damage degree of the composite specimens under various stress conditions, as well as the uneven distribution of internal micro-cracks, friction, and sliding.

**Figure 22:**
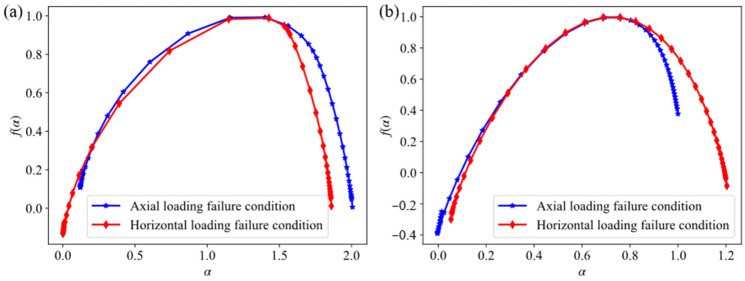
Multifractal spectrum of AE energy and rise time under different loading conditions: (**a**) AE energy; (**b**) AE rise time.

First, the AE signal energy and rise time of the entire loading process were analyzed under various stress conditions using multifractal analysis. The resulting multifractal spectrum parameters are presented in Table 2, and the corresponding multifractal spectrum is shown in Figure 22. The multifractal spectral curves obtained exhibit uniform left-skewed distribution patterns. The ∆f associated with AE energy during axial loading failure demonstrates a negative value close to 0, suggesting a relatively balanced occurrence of large energy events and small energy events. Conversely, the ∆f corresponding to AE rise time notably exceeds 0, indicative of a damage evolution process predominantly characterized by shear failure. Conversely, during horizontal loading failure, large energy events occupy a comparatively higher proportion.

**Figure 23:**
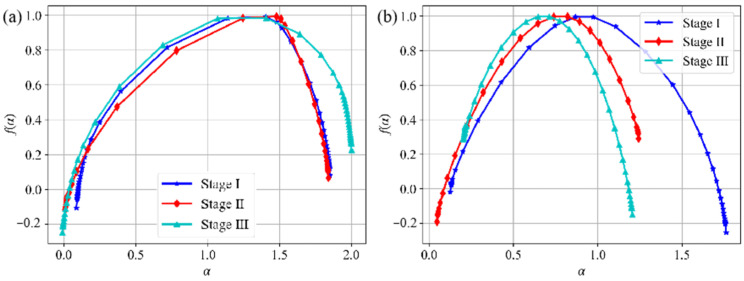
Multifractal spectrum of AE energy and rise time at different loading stages: (**a**) AE energy; (**b**) AE rise time.

Moreover, based on the variation in the AE event rate, the failure process of the composite specimen under horizontal loading is segmented into three stages. Subsequently, the AE signals at distinct stages undergo multifractal analysis for further examination, and the corresponding multifractal spectrum is shown in Figure 23. The corresponding parameter values are shown in Table 3 and Table 4.

During each loading stage, the  ∆f  values corresponding to acoustic emission energy are consistently positive, with a notable increase from Stage II to Stage III. Meanwhile, $∆\alpha_{0}\;$ consistently exhibits a negative trend, initially decreasing before rising again. In Stage I, the damage evolution of the composite specimen is primarily driven by the compression and sealing of microcracks within it. During this stage, the granite component experiences steadily increasing stress, resulting in a relatively high signal intensity due to friction between internal particles, with $∆\alpha_{0}\;$values less than 0 and  ∆f  values greater than 0. In Stage II, the damage evolution process of assembled specimens becomes more intricate. Although the  ∆f  corresponding to AE energy mirrors that of Stage I, its $∆\alpha_{0}\;$ decreases notably, suggesting that relatively minor large energy events predominantly influence the damage evolution during this stage. Multifractal analysis of AE rise time parameters indicates that shear failure signals constitute a significant portion at this stage, with those of lesser large energy playing a dominant role in the damage evolution process. In Stage III, there is a notable increase in  ∆f, with a corresponding rise in the occurrence of large energy events. However, the accompanying slight increase in $∆\alpha_{0}\;$ suggests a diminishing influence of large energy events on the damage evolution.

The parameters associated with AE rise time exhibit distinct trends. Initially,  ∆f  increases before decreasing, whereas $∆\alpha_{0}\;$ decreases before subsequently increasing. The fluctuation in  ∆f and indicates clear shifts in the predominant failure modes of the composite specimens at each stage.

## 4. Discussion

This work presents the application of two nondestructive monitoring techniques, AE and DIC, in monitoring backfill–rock combinations under different loading conditions. AE and DIC can describe the damage of backfill–rock combinations from various perspectives. AE can well compensate for the limitation of the DIC monitoring area, and its monitoring results are consistent.

The DIC results clearly observe the specimen’s damage process under various loading conditions and align with subsequent analyses of strain and deformation fields. Compared to the AE and DIC results, the AE signal exhibits higher sensitivity, showing corresponding fluctuations before notable deformation in regions observed through speckle photos, abnormal strain concentration in the strain field, and abnormal displacement in the deformation field. At the same time, the AE source location results can more clearly reflect the overall damage process of the specimen, and the distribution of AE sources at different stages has a specific reference for the internal damage location of the specimen. In order to remove the influence of AE sensors on the strain field on the DIC monitoring surface, we chose to directly remove the corresponding area during processing, but AE monitoring is full range. The strain field variation pattern of backfill–rock at the four endpoints may deviate from the AE results, which is worth further analysis and discussion.

In this work, the energy and rise time of acoustic emission signals are selected for multifractal analysis. Through the multifractal analysis of acoustic emission energy, the size of the damage to the assembly can be understood, and the multifractal analysis of acoustic emission rise time can explain the dominant fracture form.

Previous research on backfill–rock combinations primarily focused on the mechanical properties of simple structural assemblies under uniaxial compression, interface damage behavior, and the impact of backfill characteristics on the overall structure. In this work, the complex structure of backfill–rock combination is studied, which is directly related to the field conditions. When encountering abnormal axial stress, the backfill exhibits significant deformation, corresponding to the phenomenon of roof collapse in the field. When the abnormal stress increases, the surrounding rock on both sides also experiences large-scale rupture. When encountering abnormal horizontal stress, the backfill experienced significant large-scale rupture, followed by the appearance of obvious abnormal stress concentration in the surrounding rock on both sides under the influence of abnormal stress. These results are consistent with the actual field conditions.

The experiments in this work were conducted under laboratory conditions. There are several areas for improvement, including experimental design and better consideration of backfill. The loading block can be redesigned to arrange AE sensors in all directions around the specimen, creating a more comprehensive sensor network. Additionally, surrounding rock conditions and backfill vary across different engineering environments. For large-scale backfill, stratification characteristics must also be considered. Still, the field engineering environment is more complex, and the continuous action of stress is a more long-term process that needs to consider more challenges. First of all, complex and variable production operations greatly impact the long-term monitoring of AE systems. At this time, it is necessary to use microseismic monitoring equipment that is more suitable for field scales. At the same time, the position of the sensor, as well as its coupling and protection, need to be considered. Considering ambient lighting changes, DIC monitoring also requires more stable and clear equipment.

## 5. Conclusions

In the downward-approach mining method, the structural health and safety of the approach are increasingly concerning in stope safety production. Disturbances generated by blasting and other engineering operations often destabilize the surrounding rock structure, leading to roof collapses, slab tilts, or even more severe production accidents. However, the process of damage evolution in complex backfill–rock structures remains unclear. Therefore, biaxial compression tests were conducted on backfill–rock specimens using DIC and AE technology. Additionally, AE characteristic parameters are analyzed using multifractal theory. The main conclusions are as follows:

(1) The damage evolution characteristics of backfill–rock combinations vary significantly under different loading conditions. Under axial loading, the backfill undergoes significant deformation. As this deformation intensifies, the granite ultimately experiences large-scale fracturing. The backfill exhibits high residual strength during this process. Conversely, under horizontal loading failure, backfill–rock combinations exhibit cooperative bearing characteristics. As backfill parts gradually destabilize and fail, the proportion of load borne by granite increases. This leads to apparent abnormal strain concentration areas in various directions within the strain fields.
(2) The instability process of the backfill–rock combination can be categorized into three stages based on changes in acoustic emission events. The distribution characteristics of these events at various loading stages can elucidate the damage evolution process within the combination. In the initial stage, AE events are mainly concentrated in the backfill. As loading progresses to the second stage, a large number of AE events appear in the granite, consistent with DIC results. Additionally, AE events in the backfill are significantly concentrated above the void. In the third stage, the AE event distribution within the backfill becomes uniform, indicating that the damage evolves from local to overall.
(3) The multifractal degree of acoustic emission energy and rise time in backfill–rock combinations remains consistent across various loading conditions, characterized by left-skewed multifractal spectra. Axial loading conditions result in small-scale cracking and shear failure in the backfill–rock combination. Horizontal loading leads to dominant large-scale rupture and shear failure in the backfill–rock combination.

## Figures and Tables

**Table 1:** Backfill ratio of composite specimen.

Filling Material	Cement-Sand Ratio	Concentration/%
m_cement_:m_aggregate_ = 1:4 In aggregate, m_Bar scrub_:m_Gobi sand_ = 6:4	1:4	82

**Table 2:** Multifractal spectrum parameters under different loading conditions.

Loading Condition	∆α		$∆\alpha_{0}$		∆f	
	AE Energy	AE Rise Time	AE Energy	AE Rise Time	AE Energy	AE Rise Time
Axial loading	1.879	1.003	−0.630	−0.513	−0.044	0.767
Horizontal loading	1.889	1.151	−0.826	−0.261	0.155	0.215

**Table 3:** Multifractal spectrum parameters of AE energy at different stages under horizontal loading conditions.

Loading Condition	Stage I			Stage II			Stage III		
	∆α	∆f	$∆\alpha_{0}$	∆α	∆f	$∆\alpha_{0}$	∆α	∆f	$∆\alpha_{0}$
Horizontal loading	1.764	0.185	−0.872	1.834	0.186	0.475	2.012	0.475	−0.822

**Table 4:** Multifractal spectrum parameters of AE rise time at different stages under horizontal loading conditions.

Loading Condition	Stage I			Stage II			Stage III		
	∆α	∆f	$∆\alpha_{0}$	∆α	∆f	$∆\alpha_{0}$	∆α	∆f	$∆\alpha_{0}$
Horizontal loading	1.637	−0.234	0.152	1.196	0.481	−0.354	1.004	−0.434	−0.019

## Data Availability

Data are contained within the article.

## References

[1] Cai M., Wang J., Wang S. (2001). Prediction of Rock Burst with Deep Mining Excavation in Linglong Gold Mine. J. Univ. Sci. Technol. Beijing.

[2] Wagner H. (2019). Deep Mining: A Rock Engineering Challenge. Rock Mech. Rock Eng..

[3] Ranjith P.G., Zhao J., Ju M., De Silva R.V.S., Rathnaweera T.D., Bandara A.K.M.S. (2017). Opportunities and Challenges in Deep Mining: A Brief Review. Engineering.

[4] He M., Wang Q. (2023). Rock dynamics in deep mining. Int. J. Min. Sci. Technol..

[5] Yang S., Tian W., Liu X., Huang Y., Yang J. (2021). An experimental study on failure mechanical behavior and cracking mechanism of rectangular solid sandstone containing two non-coplanar fissures under conventional triaxial compression. Theor. Appl. Fract. Mech..

[6] Li X., Gong F., Tao M., Dong L., Du K., Ma C., Zhou Z., Yin T. (2017). Failure mechanism and coupled static-dynamic loading theory in deep hard rock mining: A review. J. Rock Mech. Geotech..

[7] Cao H., Gao Q., Zhang X., Guo B. (2022). Research Progress and Development Direction of Filling Cementing Materials for Filling Mining in Iron Mines of China. Gels.

[8] Yilmaz E., Belem T., Benzaazoua M. (2014). Effects of curing and stress conditions on hydromechanical, geotechnical and geochemical properties of cemented paste backfill. Eng. Geol..

[9] Feng X., Zhang Q., Ali M. (2020). 3D modelling of the strength effect of backfill-rocks on controlling rockburst risk: A case study. Arab. J. Geosci..

[10] Wang Y., Lu H., Wu J. (2021). Experimental investigation on strength and failure characteristics of cemented paste backfill-rock composite under uniaxial compression. Constr. Build. Mater..

[11] Zhao K., Huang M., Zhou Y., Yan Y., Wan W., Ning F., He Z., Wang J. (2022). Synergistic deformation in a combination of cemented paste backfill and rocks. Constr. Build. Mater..

[12] Fang K., Fall M. (2018). Effects of curing temperature on shear behaviour of cemented paste backfill-rock interface. Int. J. Rock Mech. Min..

[13] Weilv W., Xu W., Jianpin Z. (2021). Effect of inclined interface angle on shear strength and deformation response of cemented paste backfill-rock under triaxial compression. Constr. Build. Mater..

[14] Selçuk L., Aşma D. (2019). Experimental investigation of the Rock—Concrete bi materials influence of inclined interface on strength and failure behavior. Int. J. Rock Mech. Min..

[15] Yang L., Hou C., Zhu W., Li L. (2024). Effect of roughness on shear behavior of interface between cemented paste backfill and rock. Constr. Build. Mater..

[16] Xu X., Fall M., Alainachi I., Fang K. (2020). Characterisation of fibre-reinforced backfill/rock interface through direct shear tests. Geotech. Res..

[17] Xin J., Jiang Q., Liu Q., Zheng H., Li S. (2023). A shear constitutive model and experimental demonstration considering dual void portion and solid skeleton portion of rock. Eng. Fract. Mech..

[18] Xu B., Li Y., Wang S., Luo H., Lu B. (2022). Study on the strength characteristics and failure characteristics of the composite load-bearing structure in the cemented filling field. Constr. Build. Mater..

[19] Yang L., Hou C., Zhu W., Liu X., Yan B., Li L. (2022). Monitoring the failure process of cemented paste backfill at different curing times by using a digital image correlation technique. Constr. Build. Mater..

[20] Jekateryńczuk G., Piotrowski Z. (2024). A Survey of Sound Source Localization and Detection Methods and Their Applications. Sensors.

[21] Xin G., Yang G., Li F., Liu H. (2024). A Large-Scale Three-Dimensional Apparatus to Study Failure Mechanisms of Rockfalls in Underground Engineering Contexts. Sensors.

[22] Zhang J., Peng L., Wen S., Huang S. (2024). A Review on Concrete Structural Properties and Damage Evolution Monitoring Techniques. Sensors.

[23] Dong L., Zhang Y., Bi S., Ma J., Yan Y., Cao H. (2023). Uncertainty investigation for the classification of rock micro-fracture types using acoustic emission parameters. Int. J. Rock Mech. Min..

[24] Dong L., Hu Q., Tong X., Liu Y. (2020). Velocity-Free MS/AE Source Location Method for Three-Dimensional Hole-Containing Structures. Engineering.

[25] Liu L., Zhang Z., Wang T., Zhi S., Wang J. (2024). Evolution characteristics of fracture volume and acoustic emission entropy of monzogranite under cyclic loading. Geomech. Geophys. Geo.

[26] Huang L., Wu X., Li X., Wang S. (2023). Influence of sensor array on MS/AE source location accuracy in rock mass. Trans. Nonferrous Metal. Soc..

[27] Li N., Ge M., Wang E., Zhang S. (2020). The Influence Mechanism and Optimization of the Sensor Network on the MS/AE Source Location. Shock Vib..

[28] Dong L., Cao H., Hu Q., Zhang S., Zhang X. (2024). Error distribution and influencing factors of acoustic emission source location for sensor rectangular network. Measurement.

[29] Zhao Z., Yang J., Kang Y., Xiao Y. (2024). Microcrack monitoring and fracture evolution of coal and rock using integrated acoustic emission and digital image correlation techniques. Sci. Rep..

[30] Chang J., Yao Y., Liu K., Wang Y., Yan M., Bao Y. (2024). Damage assessment of concrete beams repaired with basalt fiber-reinforced polymer sheets through digital image correlation and acoustic emission. Case Stud. Constr. Mater..

[31] Machikhin A., Poroykov A., Bardakov V., Marchenkov A., Zhgut D., Sharikova M., Barat V., Meleshko N., Kren A. (2022). Combined Acoustic Emission and Digital Image Correlation for Early Detection and Measurement of Fatigue Cracks in Rails and Train Parts under Dynamic Loading. Sensors.

[32] Zhao K., He Z., Yang J., Yan Y., Yu X., Zhou Y., Zhang X., Wang J. (2022). Investigation of failure mechanism of cement-fiber-tailings matrix composites using digital image correlation and acoustic emission. Constr. Build. Mater..

[33] Zhou Y., Yin S., Zhao K., Wang L., Liu L. (2023). Understanding the static rate dependence of early fracture behavior of cemented paste backfill using digital image correlation and acoustic emission techniques. Eng. Fract. Mech..

[34] Zhang F., Zarate Garnica G.I., Yang Y., Lantsoght E., Sliedrecht H. (2020). Monitoring Shear Behavior of Prestressed Concrete Bridge Girders Using Acoustic Emission and Digital Image Correlation. Sensors.

[35] Ashraf S., Rucka M. (2023). Microcrack monitoring and fracture evolution of polyolefin and steel fibre concrete beams using integrated acoustic emission and digital image correlation techniques. Constr. Build. Mater..

[36] Zhang G., Li H., Zou C., Wang M., Wang Z. (2023). Fracture Coalescence Process Between Two Pre-existing Flaws in Granite Based on Coupling Exterior and Interior Observation Techniques. Rock Mech. Rock Eng..

[37] Kong X., Wang E., He X., Li D., Liu Q. (2017). Time-varying multifractal of acoustic emission about coal samples subjected to uniaxial compression. Chaos Solitons Fractals.

[38] Zhang H., Guo W. (2022). Acoustic Emission Waveform Characteristics of Red Sandstone Failure under Uniaxial Compression after Thermal Damage. Sustainability.

[39] Dong L., Chen Y., Sun D., Zhang Y. (2021). Implications for rock instability precursors and principal stress direction from rock acoustic experiments. Int. J. Min. Sci. Technol..

[40] Dong L., Chen Y., Sun D., Zhang Y., Deng S. (2023). Implications for identification of principal stress directions from acoustic emission characteristics of granite under biaxial compression experiments. J. Rock Mech. Geotech..

[41] Krylov A.A., Lobkovsky L.I., Ivashchenko A.I. (2019). Automated detection of microearthquakes in continuous noisy records produced by local ocean bottom seismographs or coastal networks. Russ. J. Earth Sci..

[42] Li H., Yang Z., Yan W. (2022). An improved AIC onset-time picking method based on regression convolutional neural network. Mech. Syst. Signal Pract..

[43] Wang G., He C., Liang F., Gong S. (2023). Improvement of Autoregressive Model-Based Algorithms for Picking the Arrival Times of the P-Wave of Rock Acoustic Emission. Geotech. Geol. Eng..

[44] Dong L., Bi S., Zhang Y., Hu Q., Zhu H. (2023). Arrival-Time Detection With Multiscale Wavelet Analysis and Source Location of Acoustic Emission in Rock. IEEE Sens. J..

[45] Mandelbrot B. (1982). The Fractal Geometry of Nature.

[46] Hu S., Wang E., Li Z., Shen R., Liu J. (2014). Time-Varying Multifractal Characteristics and Formation Mechanism of Loaded Coal Electromagnetic Radiation. Rock Mech. Rock Eng..

